# Assessment of Quality of Life after Adenotonsillectomy in Paediatric Patients

**DOI:** 10.22038/ijorl.2025.87895.3956

**Published:** 2025

**Authors:** Yellur Kavitha, Kandhala Poojitha, Joish Upendra Kumar

**Affiliations:** 1 *Department of ENT, SDM College of Medical Sciences and Hospital, Shri Dharmasthala Manjunatheshwara University, Sattur, Dharwad, Karnataka, India.*; 2 *Department of Radiodiagnosis, SDM College of Medical Sciences and Hospital, Shri Dharmasthala Manjunatheshwara University, Sattur, Dharwad, Karnataka, India.*

**Keywords:** Chronic adenotonsillitis, Adenotonsillectomy, Paediatric Throat Disorders Outcome Test (T-14), Paediatric quality of life, Obstructive symptoms, Infective symptoms

## Abstract

**Introduction:**

Adenotonsillectomy is one of the most commonly done surgical procedures in paediatric population. Two main indications for paediatric adenotonsillectomy are recurrent sore throat due to bacterial infection and airway obstruction due to adenotonsillar hypertrophy. This study assessed the impact of adenotonsillectomy on quality of life (QoL) in paediatric patients (3-14 years) with chronic adenotonsillitis using the Paediatric Throat Disorders Outcome Test (T-14), focusing on obstructive and infective symptoms.

**Materials and Methods:**

A, prospective, observational study was conducted in a tertiary care hospital from May 2023 for a period of 18 months. Paediatric patients of age 3-14 years with chronic adenotonsillitis who underwent adenotonsillectomy were included in this study. Paediatric Throat Disorders Outcome Test (T-14) questionnaire form was filled by patient caregivers preoperatively at the time of hospital admission and postoperatively at 6^th^ week. Paired t-test was used to ascertain and compare the values obtained.

**Results:**

60 paediatric patients of age 3-14 years (mean age 8.7 years) were included in the study, who were diagnosed with chronic adenotonsillitis and underwent adenotonsillectomy. Symptom profile in the study population revealed obstructive issues, manifested as mouth breathing (83.33%) and snoring (45.00%), and infective symptoms presented as throat pain (60%). Following adenotonsillectomy, there was significant T-14 score reductions (p=0.0001) for obstructive (91.17%) and infective (88.10%) symptoms. These outcomes validate adenotonsillectomy’s predominant role in addressing combined adenotonsillar pathology in paediatric patients.

**Conclusion:**

Adenotonsillectomy significantly enhances QoL, reduces obstructive and infective symptoms with universal improvement supporting its use in chronic adenotonsillitis.

## Introduction

 Adenotonsillar hypertrophy causes upper airway obstruction in children with varying degrees of sleep disorder breathing (SDB). SBD presents as snoring, mouth breathing, nasal congestion, restless sleep, sleep apnoea, daytime sleepiness. If left untreated / in chronic recurrent adenotonsillar pathology, child can suffer from respiratory distress, heart failure, systemic hypertension, growth retardation, neurocognitive sequelae and impaired neurobehavioral performance. Medical measures in the form of antimicrobials, adequate hydration, medications to alleviate pain and fever are useful during acute episodes. But if the episodes become chronic and recurrent, then surgery is preferred in the form of adenotonsillectomy. Hence, early diagnosis and appropriate treatment by adenotonsillectomy helps in treating these symptoms and preventing morbidity. 

## Materials and Methods

A, prospective, cross-sectional study was undertaken in a tertiary care hospital, involving 60 paediatric patients of age 3 to 14 years, who were diagnosed with chronic adenotonsillitis and underwent adenotonsillectomy between May 2023 and October 2024, after taking institutional ethics committee clearance and informed consent of care givers of the patients. All patients underwent standard pre-operative work up before undergoing adenotonsillectomy. Consent for the surgery was given by the patient’s caregivers. Paediatric patient’s caregiver filled the T-14 questionnaire preoperatively at the time of hospital admission and 6^th^ week postoperatively. The questionnaire was provided in both the English language and the local language. The validity and reliability of questionnaire was checked in local language prior to use in the study. Each question was self-explanatory and hence it was well understood by the respondents. All patients underwent adenoidectomy by curettage using St. Clair Thompson adenoid curette and tonsillectomy by dissection and snare technique using cold steel instruments. A single surgeon with ten years of experience as an otolaryngologist performed all the surgeries. Following surgery, patients were provided with necessary post-operative care and there were no cases with any form of major complications. Patients were followed up periodically at regular intervals. 

At the end of 6^th^ week post-operatively, patient’s caregivers were made to fill the T-14 questionnaire once again. Those who were unable to come for follow up, were contacted telephonically. Both pre- and post-operative scores were compared to assess the impact of the surgery on patient’s quality of life. Table 1 illustrates the T-14 questionnaire. 

**Table 1:** paediatric throat disorders outcome test (t-14):

**Sl no**	**Question**	**No problem**	**Very mild problem**	**Mild or slight problem**	**Moderate problem**	**Severe problem**	**Problem as bad as it could be**
1	Snoring loudly during sleep	0	1	2	3	4	5
2	Irregular or stopped breathing (apnoea) during sleep	0	1	2	3	4	5
3	Daytime sleepiness	0	1	2	3	4	5
4	Noisy breathing during day	0	1	2	3	4	5
5	Breathing through mouth during day	0	1	2	3	4	5
6	Problems with poor appetite, or poor eating habits (choking on food)	0	1	2	3	4	5
7	Frequent ear ache or ear infections	0	1	2	3	4	5
8	Repeated short term throat infections that last < 2 weeks	0	1	2	3	4	5
9	Constant or chronic, throat infections that last > 2 weeks	0	1	2	3	4	5
10	Many telephone calls to doctor	0	1	2	3	4	5
11	Many visits to family doctor or A&E department	0	1	2	3	4	5
12	Taking antibiotics over & over for < 2 weeks at a time	0	1	2	3	4	5
13	Taking antibiotics for >2 weeks straight	0	1	2	3	4	5
14	Missing school days due to sore throats	0	1	2	3	4	5

The initial 6 questions of the T-14 questionnaire detect obstructive symptoms whereas the later questions are targeted towards infective symptoms. 

The data was tabulated in Statistical product and Service Solutions (SPSS, version 22). Mean values and standard deviations of the values were calculated separately for both pre-operative and 6^th^ week post-operative symptoms score. 

Preoperative and postoperative symptom scores using the T-14 questionnaire were analysed with paired t-tests. 

## Results

A total of 60 paediatric patients of age 3 – 14 years were included in the study of which 36 were males (60%) and 24 were females (40%). The 60 paediatric patients were divided into three age groups: ≤5 years, 6-10 years, and ≥11 years. The ≤5 years group included 10 patients, accounting for 16.67% of the total. The 6-10 years group was the largest, with 34 patients, representing 56.67%. The ≥11 years group consisted of 16 patients, making up 26.67%. The mean age across all patients is 8.70 years, with a standard deviation (SD) of 2.72, indicating moderate variability in age distribution. The majority of patients (56.67%) fall within the 6-10 years range, suggesting that this age group is most commonly affected by the conditions leading to adenotonsillectomy in this study.The symptom profile in our study highlighted the prevalence of obstructive symptoms, with mouth breathing reported by 83.33% (n=50), snoring by 45.00% (n=27), and bilateral nasal obstruction by 6.67% (n=4). Infective symptoms, such as throat pain (60.00%, n=36) and fever with nasal discharge (1.67%, n=1), were also notable, though less frequent. Other complaints included disturbed sleep (36.67%, n=22), poor appetite (25.00%, n=15), disturbance in day-to-day activities like missing school (23.33%, n=14), difficulty swallowing (15.00%, n=9), earache (5.00%, n=3), decreased hearing (5.00%, n=3), and ear discharge (3.33%, n=2).The mean duration of symptoms in our study was 2.28 years (SD=1.92), with the largest group (36.67%, n=22) experiencing symptoms for 1-2 years. The <1 year group accounts for 21.67% (n=13), 3-4 years for 26.67% (n=16), and ≥5 years for 15.00% (n=9). The predominance of the 1-2 years group in our study aligns with the Scottish Intercollegiate Guidelines Network (SIGN) criteria used for inclusion, which require multiple episodes over 1-3 years, indicating adherence to evidence-based surgical indications (2). 

The study compared the preoperative and postoperative symptom scores using the T-14 questionnaire and analysed it with paired t-tests. For preoperative obstructive symptoms, the mean score was 11.20 (SD = 5.20). At the 6th postoperative week for obstructive symptoms, the mean drops to 1.20 (SD = 1.10), with a mean difference of 10.00 (SD Diff. = 4.73), 89.29% effect, paired t-value of 16.3763, and p-value of 0.0001 (p < 0.05), indicating a highly significant reduction. For preoperative infective symptoms, the mean score was 13.58 (SD =7.79). At the 6th postoperative week for infective symptoms, the mean is 1.62 (SD = 1.46), with a mean difference of 11.97 (SD Diff. = 6.84), 88.10% effect, paired t-value of 13.5576, and p-value of 0.0001 (p < 0.05), also highly significant. These results are shown in Table 2. These p-values (both 0.0001) confirm that adenotonsillectomy significantly improves both obstructive and infective symptoms by the 6th week, with a slightly greater effect on obstructive symptoms (91.17% vs. 88.10%).

**Table 2:** Comparison of symptoms scores at different treatment time points by paired t test

**Time points**	**Mean**	**Std.Dv.**	**Mean Diff.**	**SD Diff.**	**Paired t**	**p-value**
Preoperative obstructive	11.20	5.20				
6th week postoperative obstructive	1.20	1.10	10.00	4.73	16.3763	0.0001*
Preoperative infective	13.58	7.79				
6th week postoperative infective	1.62	1.46	11.97	6.84	13.5576	0.0001*

*P<0.05

## Discussion

The present study provides a comprehensive evaluation of the impact of adenotonsillectomy on the quality of life (QoL) of paediatric patients aged 3-14 years with chronic adenotonsillitis, utilizing the Paediatric Throat Disorders Outcome Test (T-14). 

The findings reveal significant improvements in both obstructive and infective symptoms, a 100% improvement rate across all patients. This discussion compares results with existing literature, highlighting similarities and contrasts, and explores the implications of the findings in the context of paediatric otolaryngology.

### Demographic Characteristics: Age and Gender Distribution

The age-wise distribution in our study indicated that the majority of patients 56.67% (n=34) fall within the 6-10 years age group, with a mean age of 8.70 years (SD=2.72). The ≤5 years age group comprises 16.67% (n=10), and the ≥11 age years group comprises 26.67% (n=16). This distribution aligns with studies, such as that by Hopkins et al. (2010), who reported a mean age of 7.8 years among 151 children undergoing adenotonsillectomy, with most patients aged 5-10 years (1). Similarly, Konieczny et al. (2019) found a mean age of 6.9 years (SD=3.1) in their cohort of 107 children, with a peak incidence in the 5-9 years range (3). The predominance of the 6-10 years group in our study may reflect the typical age at which chronic adenotonsillitis symptoms become pronounced, prompting surgical intervention.

### Duration of Symptoms:

 The mean duration of symptoms in our study was 2.28 years (SD=1.92), with the largest group (36.67%, n=22) experiencing symptoms for 1-2 years. The <1 year group accounts for 21.67% (n=13), 3-4 years for 26.67% (n=16), and ≥5 years for 15.00% (n=9). Comparatively, Murto et al. (2020) reported a mean symptom duration of 1.8 years (SD=1.4) in a cohort of 200 children undergoing adenotonsillectomy, with 40% (n=80) having symptoms for 1-2 years (4). The predominance of the 1-2 years group in our study aligns with the Scottish Intercollegiate Guidelines Network (SIGN) criteria used for inclusion, which require multiple episodes over 1-3 years, indicating adherence to evidence-based surgical indications.

### Symptom profile:

 Symptom profile in our study highlighted the prevalence of obstructive symptoms, with mouth breathing reported by 83.33% (n=50), snoring by 45.00% (n=27), and bilateral nasal obstruction by 6.67% (n=4). Infective symptoms, such as throat pain (60.00%, n=36) and fever with nasal discharge (1.67%, n=1), are also notable, though less frequent. Other complaints included disturbed sleep (36.67%, n=22), poor appetite (25.00%, n=15), disturbance in day-to-day activities like missing school (23.33%, n=14), difficulty swallowing (15.00%, n=9), earache (5.00%, n=3), decreased hearing (5.00%, n=3), and ear discharge (3.33%, n=2). 

These findings are consistent with the literature. Hopkins et al. (2010) reported mouth breathing in 78% (n=118/151) and snoring in 62% (n=94/151) of their cohort, with throat pain in 55% (n=83/151) (1). Similarly, Konieczny et al. (2019) found snoring in 68% (n=73/107) and throat pain in 49% (n=52/107), with rarer complaints like earache (8%, n=9/107) aligning with our low rates (5.00%) (3).

### Symptom Improvement and Quality of Life: 

 The most striking finding in our study was the significant improvement in T-14 symptom scores post-adenotonsillectomy. Preoperative obstructive scores averaged 11.20 (SD=5.20), dropping to 1.20 (SD=1.10) for obstructive symptoms at 6^th^ week postoperatively (mean difference=10.00, SD Diff.=4.73, p=0.0001). For preoperative infective symptoms, the mean score is 13.58(SD=7.79) dropping to 1.62 (SD=1.46) at 6^th^ postoperatively (mean difference=11.97, SD Diff.=6.84, p=0.0001). Both p-values (0.0001, p<0.05) indicate highly significant reductions, with a slightly greater effect on obstructive symptoms. These results strongly support the efficacy of adenotonsillectomy in enhancing QoL.

Comparatively, Hopkins et al. (2010) reported a preoperative T-14 mean of 14.2 (SD=6.8), reducing to 2.1 (SD=1.9) at 6 months post-adenotonsillectomy (mean difference=12.1, p<0.001), with 92% (n=139/151) showing improvement (1). Konieczny et al. (2019) observed a preoperative T-14 mean of 15.3 (SD=7.1), dropping to 3.2 (SD=2.4) at 5 years (mean difference=12.1, p<0.01), with 89% (n=95/107) improved (3). Our greater effect size and perfect improvement rate suggest more immediate and uniform benefits, though their longer follow-up indicates sustained gains. Puttasiddaiah et al. (2023) found a preoperative mean of 16.8 (SD=8.0), reducing to 2.5 (SD=2.0) at 3 months (mean difference=14.3, p<0.001), with 95% (n=95/100) improved (5). Our improved postoperative scores (1.20 and 1.62 vs. 2.5) and higher improvement rate (100% vs. 95%) may reflect effective surgical technique.In contrast, Paradise et al. (1984) reported less dramatic improvements in a randomized trial, with tonsillectomy reducing sore throat episodes by 65% (p<0.05) compared to controls, but only 70% (n=131/187) showed significant QoL gains (6). Their focus on infective rather than obstructive outcomes and use of a control group explain the lower improvement rate compared to our 100%. 

### Clinical Implications of Symptom profile:

 The predominance of obstructive symptoms—mouth breathing (83.33%, n=50), snoring (45.00%, n=27)—over infective ones like fever and nasal discharge (1.67%, n=1) in our study underscores the role of adenotonsillar hypertrophy in driving surgical decisions. This aligns with AAO-HNS guidelines prioritizing obstructive sleep-disordered breathing (OSDB) (7). Mitchell et al. (2007) reported snoring in 70% (n=140/200) and mouth breathing in 65% (n=130/200), with significant resolution post-adenotonsillectomy (p<0.001) (8).

### Long-term vs. Short-term Outcomes:

 Our assessment at 6^th^ week postoperative aligns with short-term outcome studies but differs from longer-term evaluations. The 91.17% and 88.10% reductions in obstructive and infective scores, respectively, are comparable to short-term findings by Hopkins et al. (2010), who noted a 12.1-point T-14 drop at 6 months (p<0.001) (1). However, Konieczny et al. (2019) tracked outcomes to 5 years, reporting a sustained 12.1-point reduction (p<0.01), suggesting durability (3). Our 100% improvement rate at 6 weeks exceeds their 89% at 5 years, possibly due to early postoperative relief before potential recurrence or adaptation. A longitudinal study by Goldstein et al. (2008) on 110 children found 85% (n=94/110) maintained QoL gains at 1-year post-adenotonsillectomy (p<0.001), with 10% (n=11/110) showing symptom recurrence (9). Our lack of longer-term follow-up limits comparison, but the significant p-values (0.0001) suggest a strong initial impact that warrants extended monitoring.

### Methodological Strengths and Limitations:

 Our prospective observational design, use of the validated T-14 tool, and adherence to SIGN criteria strengthen the findings. The paired t-test results (p=0.0001) confirm significant changes, consistent with statistical rigor in similar studies (1,3). However, limitations include the short follow-up, lack of a control group and T-14 questionnaire score obtained from paediatric patients’ caretakers which becomes potential bias due to subjective and indirect nature of the assessment. 

## Conclusion

Adenotonsillectomy positively impacts patient’s quality of life, as measured by standardized tools like T-14 questionnaire. Though adenotonsillectomy is the most common surgical procedure done among paediatric patients, the clinical improvement following surgery is not documented routinely in an objective fashion. By the application of T-14 score, this study provides objective evidence of the markedly enhanced quality of life, across age, gender, and symptom duration, supporting efficacy of adenotonsillectomy in paediatric chronic adenotonsillitis.
